# Revisiting avian ‘missing’ genes from de novo assembled transcripts

**DOI:** 10.1186/s12864-018-5407-1

**Published:** 2019-01-05

**Authors:** Zhong-Tao Yin, Feng Zhu, Fang-Bin Lin, Ting Jia, Zhen Wang, Dong-Ting Sun, Guang-Shen Li, Cheng-Lin Zhang, Jacqueline Smith, Ning Yang, Zhuo-Cheng Hou

**Affiliations:** 1 National Engineering Laboratory for Animal Breeding, Key Laboratory of Animal Genetics, Breeding and Reproduction of the Ministry of Agriculture, College of Animal Science and Technology, China Agricultural University, Beijing, 100193 China; 2 Beijing Key Laboratory of Captive Wildlife Technologies, Beijing Zoo, Beijing, 100044 China; 3 The Roslin Institute and R(D)SVS, University of Edinburgh, Easter Bush, Midlothian, EH25 9RG UK

**Keywords:** Missing gene, Avian genome, de novo assembly, Evolution

## Abstract

**Background:**

Argument remains as to whether birds have lost genes compared with mammals and non-avian vertebrates during speciation. High quality-reference gene sets are necessary for precisely evaluating gene gain and loss. It is essential to explore new reference transcripts from large-scale de novo assembled transcriptomes to recover the potential hidden genes in avian genomes.

**Results:**

We explored 196 high quality transcriptomic datasets from five bird species to reconstruct transcripts for the purpose of discovering potential hidden genes in the avian genomes. We constructed a relatively complete and high-quality bird transcript database (1,623,045 transcripts after quality control in five birds) from a large amount of avian transcriptomic data, and found most of the presumed missing genes (83.2%) could be recovered in at least one bird species. Most of these genes have been identified for the first time in birds. Our results demonstrate that 67.94% genes have GC content over 50%, while 2.91% genes are AT-rich (AT% > 60%). In our results, 239 (53.59%) genes had a tissue-specific expression index of more than 0.9 in chicken. The missing genes also have lower Ka/Ks values than average (genome-wide: Ka/Ks = 0.99; missing gene: Ka/Ks = 0.90; t-test = 1.25E-14). Among all presumed missing genes, there were 135 for which we did not find any meaningful orthologues in any of the 5 species studied.

**Conclusion:**

Insufficient reference genome quality is the major reason for wrongly inferring missing genes in birds. Those presumably missing genes often have a very strong tissue-specific expression pattern. We show multi-tissue transcriptomic data from various species are necessary for inferring gene family evolution for species with only draft reference genomes.

**Electronic supplementary material:**

The online version of this article (10.1186/s12864-018-5407-1) contains supplementary material, which is available to authorized users.

## Background

Gene gain and loss are common events during various speciation processes [1]. However, high-quality genomes are an essential prerequisite for inferring gene gain and loss at the genome-wide scale. There has long been debate as to whether birds have less genes than mammals. Many genes were not found in the first avian reference genome (chicken, *Gallus gallus*), and the gene loss and/or accelerated gene evolution hypothesis in the avian lineage was proposed [2]. When more avian genomes became available, Zhang et al. [3] and Lovell et al. [4], using multiple genome comparisons, proposed there were 640 and 274 protein-coding genes (respectively) that were lost in the avian lineage. The two studies have drawn similar conclusions that these gene losses are due to fragmentation or deletion of syntenic blocks during evolution [3, 4]. However, several recent genome-wide and/or case studies recovered some genes initially presumed lost in bird genomes [5–7]. It was thought that both GC composition and GC repeats within these missing genes were significantly higher than that of other genes [5], and that they also clustered in GC-rich regions [6]. As PCR amplification is sensitive to extreme GC-content variation, this creates uneven genomic representation within classical Illumina libraries and large genomes are generally inefficiently assembled, particularly those created following standard protocols [8]. Searches for several genes that have been shown to be important in mammals but were considered to be lost in the chicken, have in fact discovered full length cDNAs for these genes [6, 9, 10]. At the time, the newly released chicken genome, Galgal5, included around 1900 protein-coding genes not present in Galgal4, annotating some of the genes previously thought to be missing [11]. Recent advances suggest that a considerable number of the presumed ‘missing genes’ are not really missing in the avian genome. As more genes are recovered, a recent study concluded that avian genomes contain similar numbers of genes to mammals and non-avian reptiles [7]. To be able to directly address these conflicts, we need strong evidence to find these missing genes in multiple bird species. Different studies have shown that recovering genes through transcriptome assembly methods is an effective method that can compensate for the impact of poor genome quality.

This study used multiple transcriptomic data sets from 5 bird species (chicken, *Gallus gallus;* duck, *Anas platyrhynchos;* pigeon, *Columba livia;* goose, *Anser cygnoides;* zebra finch, *Taeniopygia guttata*) to exhaustively searching for the missing genes in birds, and also elucidate the effects of GC content, expression pattern, and assembled genome quality on gene loss studies. We demonstrate that de novo assembly of multiple transcriptomes from various tissues can rescue most missing genes in the absence of complete reference genomes, and most presumed missing genes have a strong tissue-specific expression pattern.

## Methods

### Animal tissues and RNA-Seq

Chicken RNA-seq data encompassing 26 tissues were downloaded from GenBank. From the public dataset, we only kept the paired-end reads of at least 70 bp in length for use in the de novo assembly. Duck samples (both adult and embryos) were obtained from Pekin Gold Duck Inc. Pigeon samples were obtained from Beijing Sunyi pigeon farm. Four tissues from geese were obtained from Zhejiang Goose farm, while other tissues were download from GenBank. Zebra finch samples were obtained from the Beijing Zoo (Additional file1: Table S1). Tissue samples were snap-frozen in liquid nitrogen and then stored at − 80 °C until RNA extraction. RNA was extracted by homogenization at low temperature and preservation in Trizol reagent (Invitrogen, USA). Approximately 10 μg of sheared cDNA was prepared for Illumina sequencing according to the manufacturer’s protocols. Libraries were prepared from a 200–230 bp size-selected fraction following adapter ligation and agarose gel separation. The library was sequenced using a multiplexed paired-end protocol with 150 bp of data collected per run on the Illumina Hiseq 2500/4000. Base calling was performed by the Illumina instrument software. The FASTX Toolkit (−v 0.0.14) (http://hannonlab.cshl.edu/fastx_toolkit/) was used to filter the obtained data. Reads less than 70 bp were removed as were reads having > 5% low quality bases (<Q30).

### De novo transcriptome assembly and quality evaluation

**Fig. 1:**
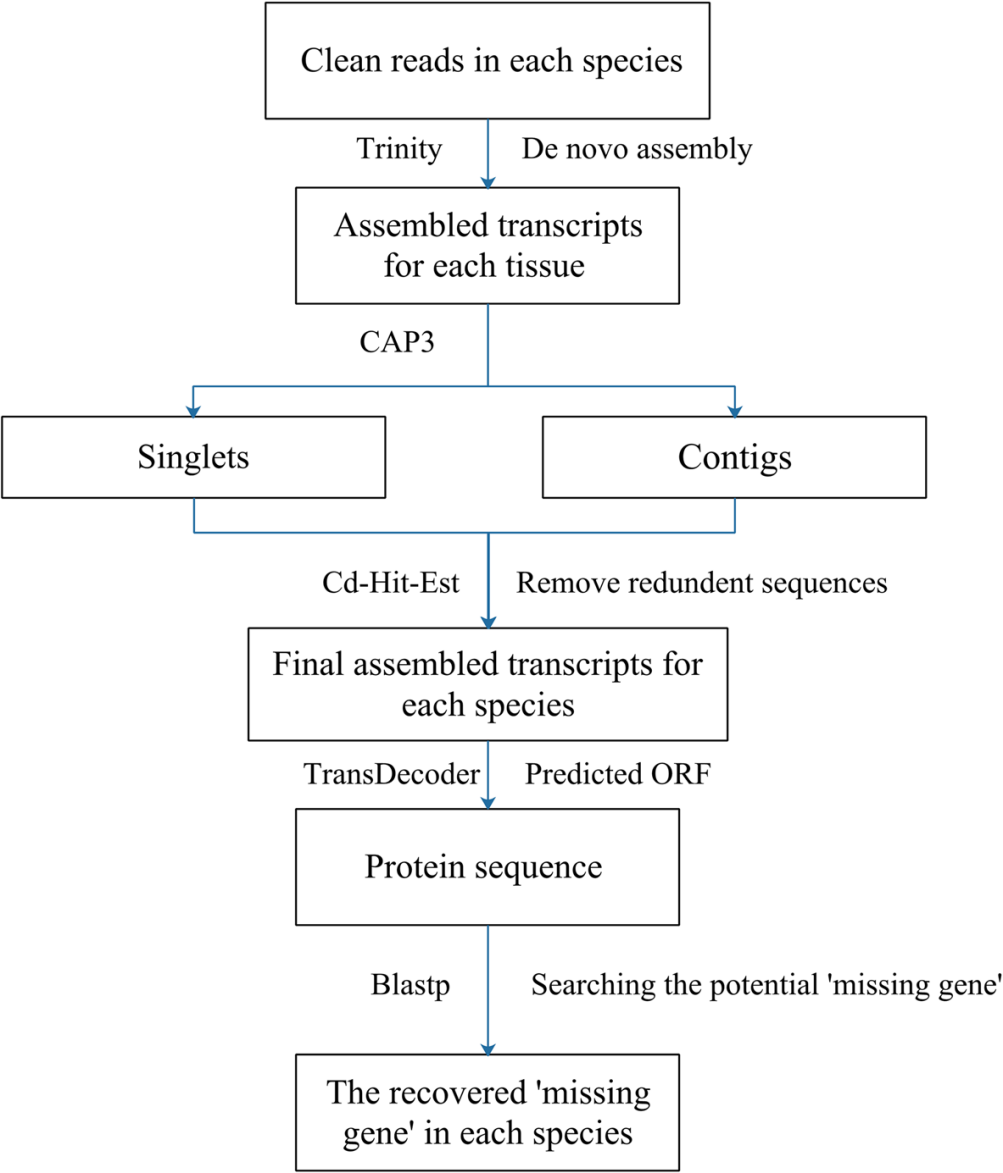
Analysis pipeline showing how to get high quality transcriptome datasets and identify “missing genes”

The analysis pipeline for discovering ‘missing genes’ is shown in Fig. 1. The analysis details are described as follows. In order to make the transcriptome as complete as possible, we assembled all RNA-Seq data that met the assembly conditions. Because the amount of data used for de novo assembly is very large, (especially in some tissues of chickens that contain multiple transcriptomic data) in order to reduce the computational memory requirements, we first used Trinity software (v2.3.2) [12] with default parameters (k-mer = 25) to assemble transcripts for each tissue in each species. CAP3 [13] was then used to assemble transcripts from different tissues into longer contigs (−c 5 -t 30). Finally, the transcripts from all tissues in each species were combined together and the redundant sequences removed using CD-HIT (−c 0.95 - aS 0.8) [14]. In order to improve the accuracy of the alignment results and reduce problems caused by assembly error, the ORF of the transcript sequence was predicted and extracted by TransDecoder (https://transdecoder.github.io) for each species. Sequences with no open reading frame were omitted. The longest transcribed sequence with an open reading frame was used for downstream analysis.

In order to ensure the accuracy of the downstream analysis, we performed a quality assessment of the assembled transcripts. We used the orthlog hit ratio (OHR) [15] to evaluate the integrity and richness of the transcripts. By comparison of the constructed sequences with the known sequences in the related species database, we defined the ratio of the best comparison results to the reference sequence of OHR. The closer the OHR is to 1.0, the more complete the constructed transcript is. The chicken has a large number of gene sequences that are well annotated, so we selected the protein-coding genes in chicken as reference sequences (Ensembl, V92). The OHR of the five species were calculated as the ratio of the length of the best CDS sequence to that of the known genes. The OHR distribution diagram of a known sequence was made using the R package (//www.R-project.org/).

### Comparative genomic analysis

Previously published candidate missing gene lists by Lovell et al. (Additional file 1: Table S1) and Zhang et al. (Additional file 1: Table S10), were used as the targets to test whether these presumably missing genes are really lost in birds. There are 274 missing genes in birds in the Lovell study and 640 genes in the Zhang results. We combined each missing gene list to obtain 806 candidate missing genes in birds. All following comparative genomics and expression studies were conducted based on this missing gene list. After obtaining the peptide sequences of these missing genes from human, we used these human genes as targets with which to search for homologous bird genes from our assembled transcripts. The BLASTP [16] program (identity> 40%; −E value = 1e-10) was used to search the bird sequences. We chose the amino-acid sequence of human orthologues to search for the orthologues from the assembled transcripts in the five bird species. Only the contig which had the highest alignment score was selected as the best candidate missing sequence in each bird. After obtaining the best sequence of the missing gene in birds, basic information such as length and GC content were calculated.

Human (*Homo sapiens*, GRCh38.p12), mouse (*Mus musculus*, GRCm38.p6) and anole lizard (*Anolis carolinensis*, AnoCar2.0) gene annotations (Ensembl V92) were used as references with which to compare the distribution of GC-content within protein-coding genes from the five birds used in this study. Co-linear analysis of chromosome fragments among human, chicken and lizard was done using LASTZ (−-step 10,--gapped) (-V 1.04) [17]. The visual map of the common linear region was made using the R package. BLASTX was used to compare recovered bird missing gene transcripts with SWISS-prot protein sequences. tBLASTn was used to compare human homologous protein sequences with Chicken (Galgal5), duck (BGI_1.0), goose (AnsCyg_PRJNA183603_v1.0), pigeon (Cliv_1.0) and Zebra finch (Taeniopygia_guttata-3.2.4) genomes.

In order to compare Ka/Ks values of missing genes with all annotated protein-coding genes in the chicken genome, we used chicken-human orthologues as references. Chicken-human single copy orthologues (Ensembl V92) were extracted using Ensembl Biomart for Ka/Ks analysis. First, the cDNA sequences were translated into amino-acid sequences and aligned by MUSCLE software [18], the aligned amino-acid sequences were converted to cDNA alignment according to the original cDNA sequences. Ka/Ks values were calculated for each orthologous group using KaKs calculator (verion 2.0) [19] with default parameters(−c = standard code, −m = MA).

### Expression analysis

Salmon software [20] was used to obtain quantitative information for each transcript sequence, including the normalized TPM and the number of reads mapped on each transcription group by default parameters. The RPKM [21] of each transcription group sequence was then calculated, and used to calculate the specific expression index of the downstream tissue. The Tissue Specific expression index (TSI) was proposed by Yanai [22], and can accurately measure the specific expression of a gene. We calculated the tissue-specific-index of high confidence genes in four species, not include goose. For TSI to be computationally significant, the number of tissues to be included should be > 10. Only 8 goose tissues were available and were thus excluded from the analysis. Genes were defined as being highly expressed in a tissue if they had expression 3-fold higher than the average expression in all tissues. We calculated the tissue-specific expression indices of genes in four species of birds - chickens, ducks, pigeons, and zebra finch as these species have data from more tissues.

### RT-PCR for candidate genes

In order to confirm the de novo assembled cDNA for some very important ‘missing genes’, we used RT-PCR and Sanger sequencing to obtain the candidate missing gene cDNA sequences. From the high-confidence gene list, we did literature searching using the missing gene name. Based on the PubMed search results, we chose those genes for which there have been in-depth studies in human, but with no related studies in birds. We used chicken-related tissues based on gene expression pattern for further RT-PCR analysis.

Total RNA was extracted from the corresponding tissue using Trizol reagent (Invitrogen, USA). First-strand cDNA was generated from 1μg of RNA using PrimeScript™ RT reagent Kit with gDNA Eraser (Takara, Japan) following the manufacturer’s instructions. Each gene-specific primer was designed using primer 5 software and the corresponding fragment was amplified in a 30 ul PCR reaction containing 1 ul cDNA, 2 mM MgCl_2_, 0.5 mM of each primer and 0.5 X super fidelity PCR mix (NEB, England). Temperature cycles were as follows: initial denaturation at 95 °C for 3 min; 30 cycles at 95 °C for 1 min; annealing at 60 °C for 20 s; polymerization at 72 °C for 1 min; and final extension step at 72 °C for 10 min. The annealing temperature and extension times varied depending on the primer Tm and the length of the fragment being amplified. Specificity of the amplification products was verified by electrophoresis on a 0.8% agarose-gel and by Sanger sequencing.

## Results

In this study, we collected publically available transcriptome data for five birds along with our own set of 87 sequenced transcriptomes. After quality control, a total of 196 transcriptome datasets from 5 birds were obtained, which comprised 3651.41GB useable data in total (Additional file 1: Table S1). This is the data used in the following analysis. The data covered almost all important tissues/organs in 5 different bird taxa. Raw transcript numbers ranged from 1,264,301 (Goose) to 2,479,109 (Zebra finch), while N50 ranged from 595 bp (Chicken) to 1533 bp (Pigeon) (Table 1). After CD-HIT and TransDecoder analysis for raw assembled transcripts, the following numbers of transcripts for each species remained: 352,401 (chicken), 350,367 (duck), 255,417(pigeon), 246,419 (goose) and 418,441(zebra finch). Quality evaluation of transcript assembly showed that most orthologue hit ratios are close to one, which indicates a high assembly quality [15] (Additional file 2: Figure S1).

**Table 1:** Summary of RNA-Seq samples and de novo assembly statistics

Species	Total Tissue Numbers	Total Clean Reads(M)	Assembled Transcripts Numbers	Assembled Transcripts N50 (bp)
Chicken	26	7353	2,048,631	596
Duck	24	2282	2,012,592	656
Pigeon	11	904	1,491,614	1533
Goose	8	708	1,264,301	1004
Zebra finch	22	1372	2,479,109	965
Total	**99**	**12,619**	**9,296,247**	

Total clean reads (M): millions of paired-end reads for each tissue

**Fig. 2:**
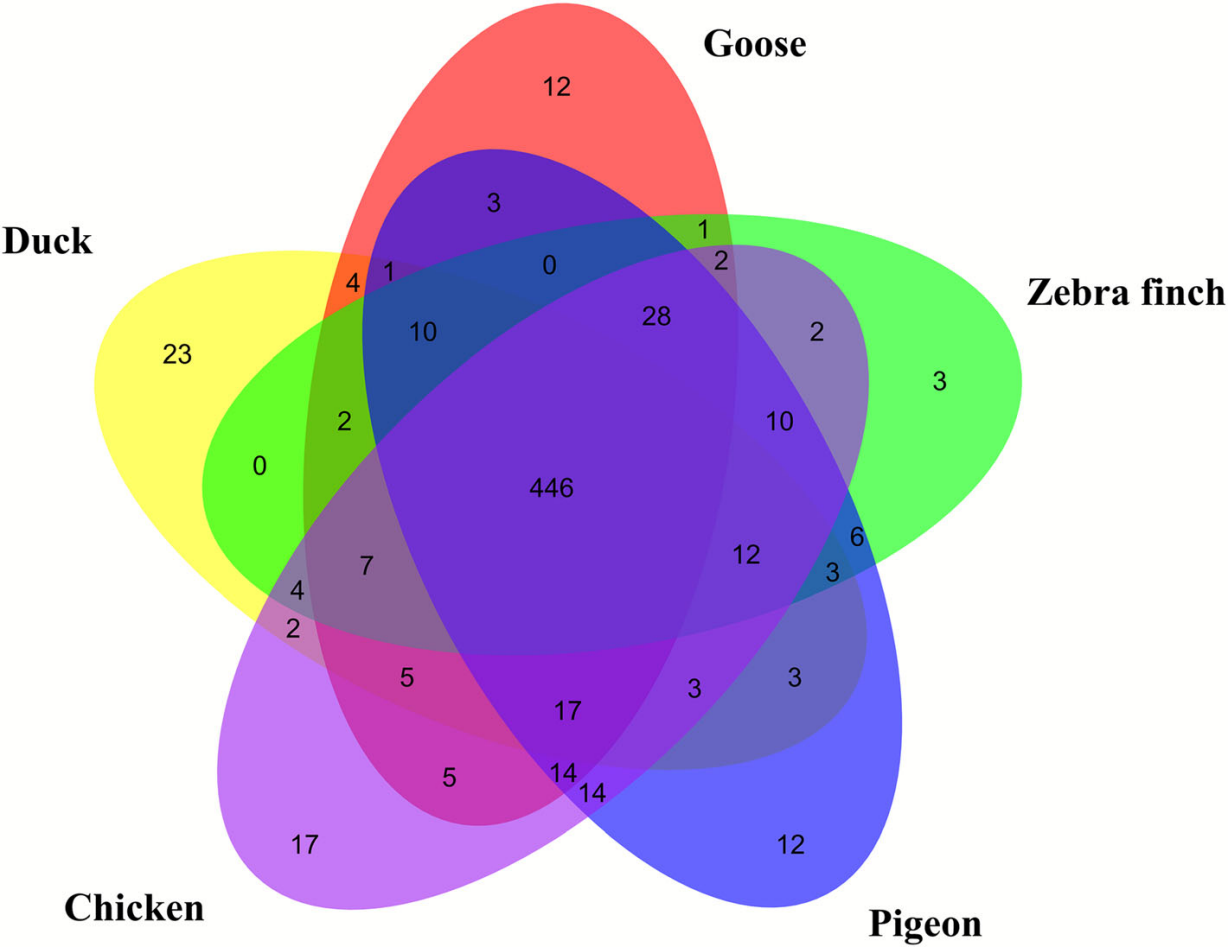
Venn diagram of recovered ‘missing’ genes in each species. High-confidence recovered ‘missing’ genes from five species (chicken, duck, goose, pigeon and zebra finch). Most of the ‘missing’ genes were recovered from all five species

We exhaustively searched the missing genes described by Zhang et al. [3] and Lovell et al. [4] that were thought lost in the bird genome (Additional file 1: Table S2). We used blastp [16] (e value = e-10, identity% > 40) to align human homologous protein sequences to the translated protein sequences from the five assembled bird transcript datasets. According to the comparison results, the recovered missing genes were classified into three bins: high-confidence genes (recovered in all five species), medium-confidence genes (recovered in three to four birds), low confidence genes (existing in one or two species). The recovered missing genes from five birds were 589 (chicken), 583 (duck), 537 (goose), 558 (pigeon), and 543 (zebra finch) (Additional file 1: Table S3A) from the missing genes list. Of these, 446 (446/807 = 55.27%) were high-confidence genes which means they were found in all five species (Fig. 2), while the medium-confidence bin included 118 genes, with 107 genes falling into the low-confidence bin. In total, most of these missing genes (671/806 = 83.25%) were found in at least one bird species (Additional file 1: Tables S3A).

We mapped high-confidence chicken, duck, goose, pigeon and zebra finch transcripts to their corresponded reference assembly. This comparison found that all these genes could only be very partially mapped onto the reference genome (Additional file 1: Table S4). Meanwhile, we also used human homologous protein sequences from the missing gene list to compare with the 5 bird genomes using tblastn [16] (−E value = e-10). This yielded 556 (chicken, Galgal5), 513 (duck), 506 (goose), 529 (pigeon), and 495(zebra finch) matches (Additional file 1: Table S5). The alignment quality of the de novo assembled transcripts is much better than using human protein sequences (Additional file 1: Table S4, S5). All these results confirm the wide existence of presumed missing genes in the five birds studied.

**Fig. 3:**
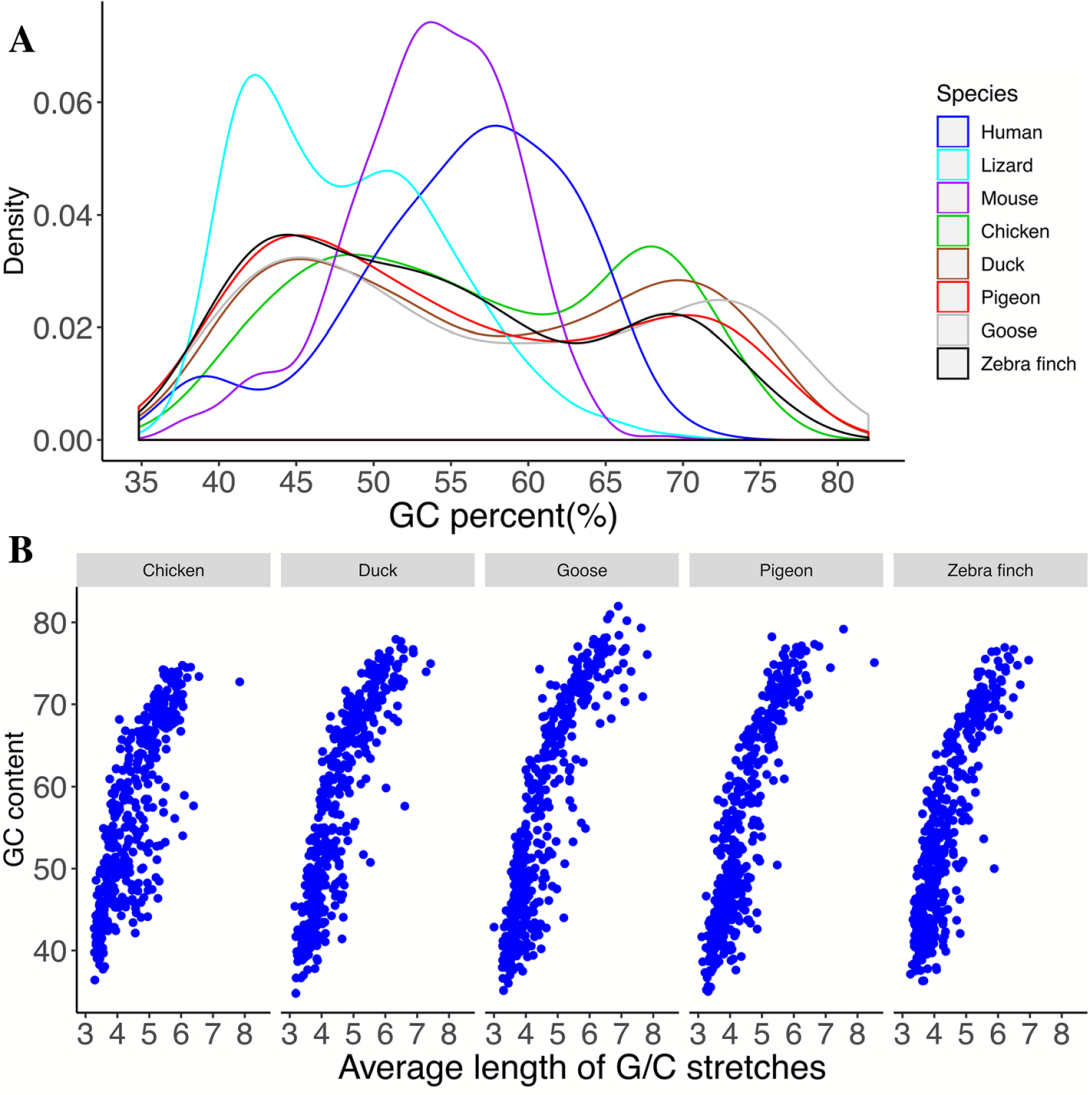
GC-content and GC-repeats in high-confidence genes. **a** GC content of high-confidence genes in five bird species (chicken, duck, pigeon, goose, zebra finch). This figure shows GC content distribution of missing genes in five bird species and representatives of non-avian animals (human, mouse and anole lizard). **b** This figure shows the relationship of GC content and GC repeats in the five birds

Recent studies have suggested that high GC content is one reason for being unable to find certain genes within a genome [6, 7, 23]. The overall GC content of these genes is relatively high (GC = 66.99%, Additional file 1: Table S6), similar to previous findings [6]. Among the 446 high-confidence genes recovered in this study, 29.37% (*n* = 131) have a GC content of 40–50%, and 302 (67.94%) have GC content over 50%. There are 13 genes (2.91%) which are AT-rich (AT% > 60%; Fig. 3a**;** Additional file 1: Table S6). The medium and low-confidence gene sets show similar trends with GC content distribution. About 60% of genes are GC-rich, with the rest being comparable with genome background. Average GC content of the discovered gene set is 56.72% which is significantly higher than the genome-wide chicken transcriptome (*P* = 2.2E-16, t-test). We found that the average GC content of these missing genes is higher than other annotated coding genes, although not reaching an extreme level. We also analyzed the GC content of high-confidence genes in different species and found that the average GC content ranged from 51.23% (lizard), through birds (zebra finch, 54.26%; goose, 56.90%), to 59.57% (human) (Additional file 1: Table S6). Interestingly, GC content distribution of the ‘missing’ genes has a similar bimodal distribution pattern in birds (Fig. 3a). Further analysis revealed that GC-stretches for most of the high-confidence genes would be expected, and we did not observe long GC fragment repeats in birds (Fig. 3b).

Due to the presence of microchromosomes in birds, the avian genome is seen to represent a highly stable karyotype [2, 7]. As we have now recovered those ‘missing’ genes in our five studied birds, we can re-analyze the chromosomal location of these genes to investigate whether there are indeed lost syntenic blocks. Among the mapped 419 high-confidence transcript sequences on the Galgal5 chicken assembly, 322 (76.85%) gene sequences aligned to known chromosomes and 91 (21.72%) gene sequences mapped to unplaced scaffolds (Additional file 1: Table S4; Additional file 2: Figure S2). We directly performed a co-linear analysis of the corresponding human, chicken, and lizard chromosomal segments of the four syntenic blocks (Additional file 2: Figure S3) which harbor the relatively closely-linked missing genes, and found that these regions were partially homozygous. The other mapped genes distributed on different chromosomes/un-placed contigs, with no obvious clustering (Additional file 1: Table S7)**.**

**Fig. 4:**
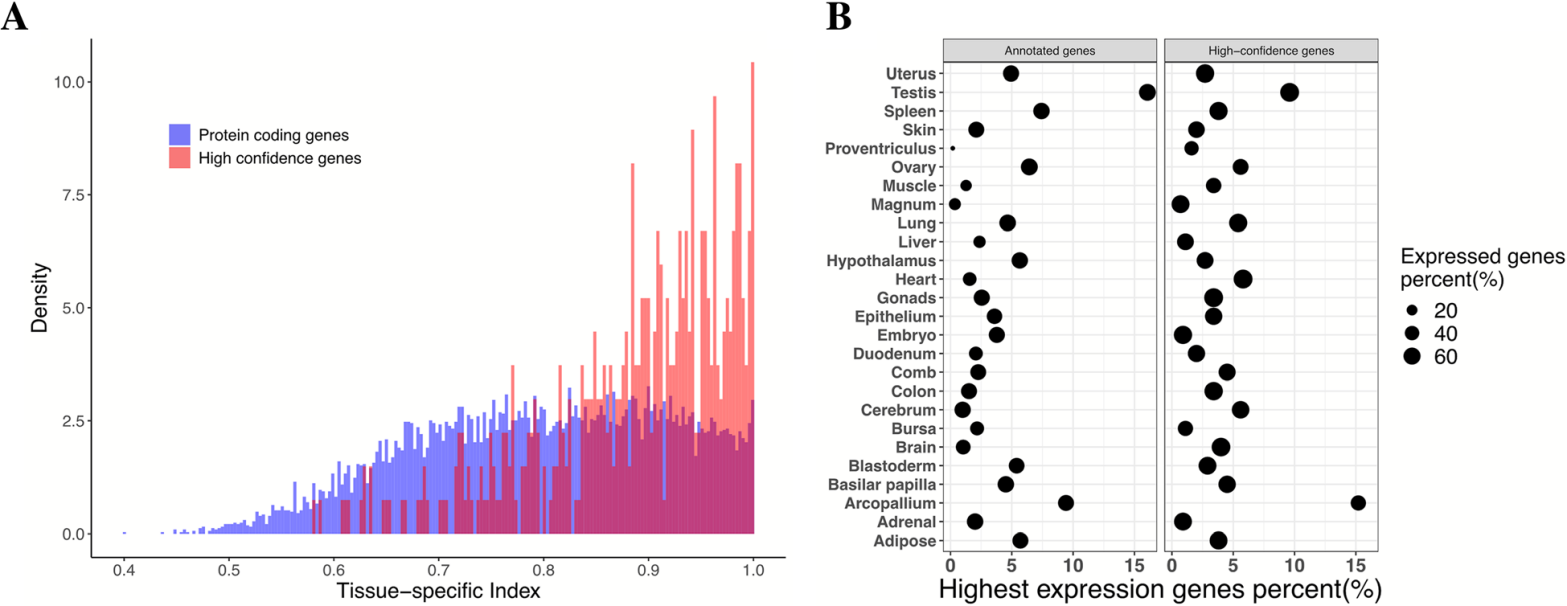
Expression pattern of high-confidence genes and annotated genes. **a** Tissue-specific expression index (TSI) of high-confidence genes in chicken. TSI of high-confidence genes is significantly higher than genome background. **b** The percentage of genes that expressed most highly in each tissue and the percentage of expressed genes in each tissue both in high-confidence genes and annotated genes. In all chicken tissues, the percentage of expressed genes in high-confidence genes is significantly lower than in annotated genes

We investigated the expression pattern and calculated the tissue-specific expression index [24] for each gene in the five species. Of all the reconstructed high-confidence genes, 239 (53.59%) had a tissue-specific expression index of more than 0.9 in chicken, which is significantly higher than the genome-wide average (average TSI genome-wide = 0.79, average TSI for missing genes = 0.89, t-test = 2.2E-16) (Fig. 4a). These missing genes not only have a very strong tissue-specific expression pattern in birds but are also lowly expressed in most tissues (Additional file 1: Table S8). Based on our data, we compared the missing gene expression pattern and the current known gene model using the ratio of highest expression gene/total gene numbers in each category. There are several tissues, i.e., arcopallium, lung and gonads which are enriched for more missing genes compared with known gene models. There are several tissues, i.e., uterus, testis and adipose, which are enriched for highly expressed missing genes (Fig. 4b**;** Additional file 2: Figure S4). These tissues would be good candidates for choosing to explore more new gene models when using transcriptome-based de novo assembly methods.

**Fig. 5:**
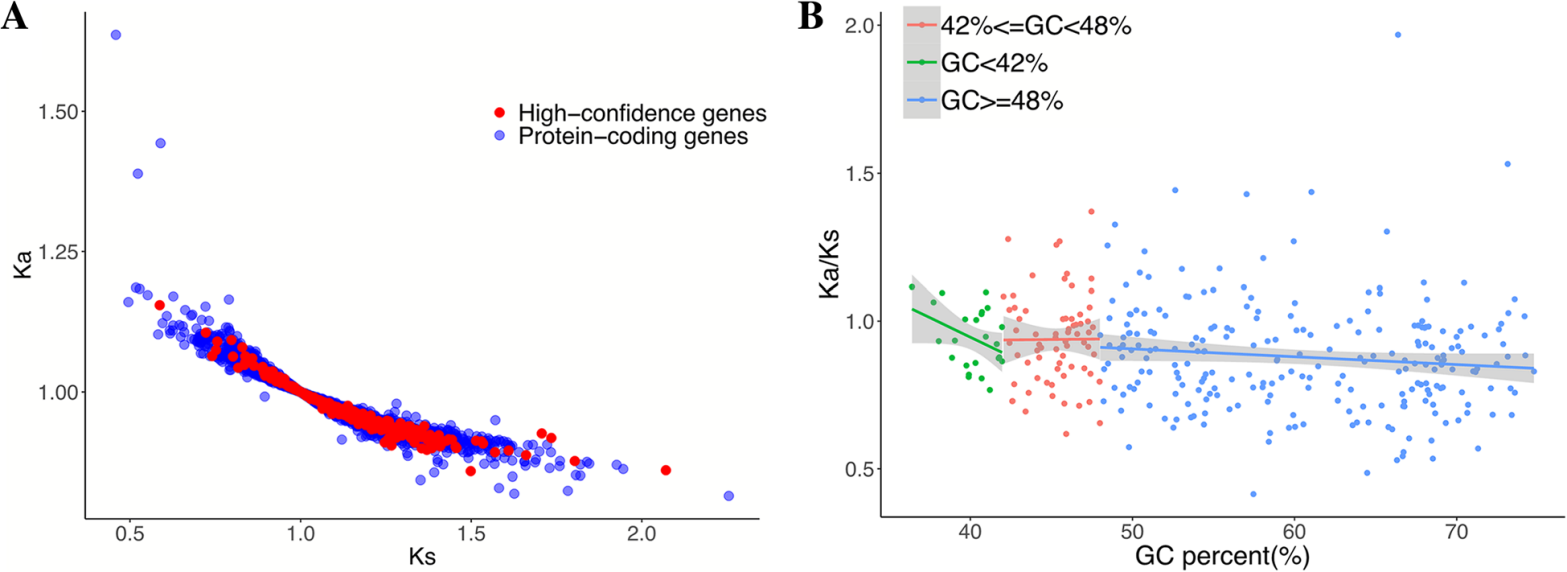
The relationship between GC percentage and Ka/Ks in high-confidence genes and genome-wide genes. **a** Ka/Ks comparisons of high-confidence genes and genome-wide genes. **b** This figure shows a higher dispersion pattern as the GC-content increases while average Ka/Ks values decrease as the GC-content increases

To investigate whether these ‘missing’ genes are evolving faster than other genes, the human-chicken single copy orthologues were identified and the Ka/Ks values calculated. These were then systematically compared to the genome-wide average. The results showed that the missing genes have lower Ka/Ks values than average (genome-wide: Ka/Ks = 0.99; missing gene: Ka/Ks = 0.90; t-test = 1.25E-14) (Fig. 5a), indicating that most presumed missing genes have undergone stronger purifying selection. We found that Ka/Ks values showed a higher dispersion pattern as the GC-content increases while average Ka/Ks values decrease as the GC-content increases (Fig. 5b). In general, functionally important genes have undergone stronger purifying selection than non-functionally important genes [25]. Missing genes are generally more conserved compared to the genome average, which might suggest functional importance for some of these missing genes. We did literature searching for the recovered missing genes in humans, and used the number of hits as one indicator of importance (Additional file 1: Table S9). Previous studies [6] and our results suggest the importance of some missing genes in birds. Both the Zhang and Lovell studies show that some of these genes are known to be important in mammals. From existing biological knowledge, we selected 10 genes (*CNOT3, HCFC1, KDM6B, PTGIR, GNG8, SLC7A8, CEBPE, RASSGRP4, FBXL19, BCL7C*) from the putative missing gene list and performed QRT-PCR for confirmation along with more detailed analysis. There have been in-depth studies on these genes in human, but there are no related studies in birds. All 10 genes were successfully cloned, their sequence verified (Additional file 1: Table S9; Additional file 2: Figure S5-S15) and their presence confirmed in the bird genomes. Our study can recover most presumably- lost genes in birds which can be inferred from comparison of avians with other vertebrates. These results clearly show that avian species have not lost very many genes when compared with other vertebrates.

## Discussion

In this study, we found that a small portion of missing genes don’t have genomic/transcripts information based on current reference assembly and de novo assembled transcripts. After exhaustively searching de novo assembled transcripts and their current reference genomes for all five birds, we could not find any orthologues for 135 genes in any assembled transcriptome from the five birds, and didn’t find meaningful orthologues in any of the five bird reference genomes. These results suggest that these 135 genes are most probably lost in avians (Additional file 1: Table S3B). All the missing genes described by Bornelov et al. [6] who reconstructed chicken transcripts from transcriptome data from three tissues, were also found in our assembled chicken transcripts, of which 34 were found in all 5 birds (Additional file 1: Table S3A). To determine whether these 135 genes are really missing in birds, will require further studies. Furthermore, precisely inferring these missing genes also depends on multiple finished bird genomes.

Recent studies combined both mapping-based annotation and de novo assembly methods to predict chicken transcripts, and obtained 20% more transcripts than the ENSEMBL annotation pipeline [26]. By comparing the newly constructed chicken high-confidence transcript sequence with two different chicken reference genomes (Galgal5, Galgal4), we obtained 419 and 382 alignments, respectively (Additional file 1: Table S4). Thirty-four genes missing in Galgal4 were also found annotated in the improved GalGal5 assembly (Additional file 1: Table S10). Our study also found all high-confidence genes were also present in the different bird reference assembly (Additional file 1: Table S4). This result helps explain why current genome annotation does not include these genes. Both genome assembly and annotation have major impact on inferring missing genes. As the quality of the genome assemblies improve, the numbers of genes in birds will increase.

Based on current results, it was found that high GC content was only one cause of missing genes in general. It is observed that GC content of these missing genes is slightly increased from lizard through to human. The evolutionary significance of this change in genic GC content is something that should be revisited. Previous studies have shown that microchromosomes harbour higher gene-density, GC content and recombination rates than macrochromosomes [27]. Recombination is tightly related to the phenomenon of GC-biased gene conversion [28]. These high GC-content missing gene are actually present in the avian genome, and had also been hypothesized as being part of missing blocks of genes [4]. The majority of the missing genes were recovered in the microchromosomes and unplaced scaffolds. The process of GC-biased gene conversion (gBGC) has a major impact on recombination rate across the genome [29, 30]. The gBGC may play a major role in the high recombination rates seen in avian microchromosomes.

Our results also found very interesting results that current missing genes are highly enriched in the tissue-specific expressed group. Unique tissue specificity and low expression of genes are some of the reasons that hinder the construction of high quality transcripts using RNA-Seq data. In this study, more than 55% (high-confidence) or 88% (low-confidence) of the proposed missing genes were obtained through assembly of 196 transcriptomic data sets, indicating that multi-tissue transcriptome assembly can largely solve the missing gene problems caused by poor genome quality. This a good complementary strategy for concluding gene loss in the absence of very-high quality genomic/annotation data.

## Conclusions

We constructed a relatively complete and high-quality bird transcript database from a large amount of avian transcriptomic data, and recovered most of the genes previously presumed to be missing in birds. Most of these genes have been identified for the first time in birds, and some incorrectly annotated genes were also corrected. From our comprehensive analysis results, we can demonstrate that detailed transcriptomic data from various tissues/organs are an essential complement to inferring gene gain and loss, before we can achieve a ‘finished’ genome. Based on the current study, we conclude that most of the presumed missing genes are in fact present in the bird genomes, but not in the current reference assemblies. High GC-content is one reason for wrongly inferring missing genes in birds, and some of these genes (about 40%) have similar, or lower, GC-content compared with genome background. Those presumably missing genes often have a very strong tissue-specific expression pattern. This study demonstrates that high quality genome data and annotation are necessary for investigating true gene loss.

## Additional files

Additional file 1: Description of RNA-Seq data sets, recovered missing genes, mapping, expression related data. **Table S1.** Overview of RNA-Seq data used in this study. **Table S2.** List of missing genes investigated in this study. All genes were from Zhang et al. [3] and Lovell et al. [4]. **Table S3.** Missing genes recovered by analysis of five bird transcriptomes. **Table S3A.** lists the evidence supporting the presence of missing genes as described both in Zhang et al. [3] and Lovell et al. [4]; **Table S3B.** list the genes which have not been recovered and appear to be truly absent from the avian genome. **Table S4.** Mapping information of recovered missing genes in the bird genomes and homologs in SWISS-prot. **Table S5.** Missing gene tBLASTn against the bird genomes. Results of human homologous protein sequences blasted against the 5 bird genomes. **Table S6.** GC-content of high-confidence genes in five birds. **Table S7.** Co-linear analysis of presumed missing blocks in chicken and other animals. **Table S8.** Tissue-specific expression index of high-confidence genes. **Table S9.** The number of related studies of 446 high confidence genes in PubMed. Searchers were carried out using official gene symbols. **Table S10.** List of 34 new annotated missing genes in the chicken genome (Galgal5). We used the missing gene list to search the new annotation from the Galgal5 reference compared with Galgal4 reference. (XLSX 590 kb)

Additional file 2: Details of sequence validation and comparative genomics information. Table S11. List of primers used for PCR validation. **Figure S1.** Distribution of orthology hit ratio of assembled transcripts using chicken annotation (Galgal5) as a reference with which to compare de novo assembled transcripts. This figure shows that most assembled transcripts are close to the reference annotation, which represents the high quality of assembled transcripts. **Figure S2.** Distribution of recovered missing genes on chicken chromosomes. **Figure S3.** Co-linear analysis of chicken-human missing blocks. **Figure S4.** The comparison of gene expression pattern between high confidence genes and annotated genes in chicken. **Figure S5-S15.** The alignment of validated genes (*CNOT3, HCFC1, KDM6B, PTGIR, GNG8, SLC7A8, CEBPE, RASSGRP4, FBXL19, BCL7C*). (DOCX 2489 kb)

## Abbreviations

RPKM: Reads Per Kilobase of exon model per Million mapped reads
TPM: Transcripts Per Kilobase of exon model per Million mapped reads
TSI: Tissue-specific expression index
