# Supplementary material for: Revisiting avian ‘missing’ genes from de novo assembled transcripts
Source: BMC Genomics. 2019 Jan 5;20:4. doi: 10.1186/s12864-018-5407-1 (PMC6321700; doi:10.1186/s12864-018-5407-1)
Supplement: Supplementary file 2 — Details of sequence validation and comparative genomics information. Table S11. List of primers used for PCR validation. Figure S1. Distribution of orthology hit ratio of assembled transcripts using chicken annotation (Galgal5) as a reference with which to compare de novo assembled transcripts. This figure shows that most assembled transcripts are close to the reference annotation, which represents the high quality of assembled transcripts. Figure S2. Distribution of recovered missing genes on chicken chromosomes. Figure S3. Co-linear analysis of chicken-human missing blocks. Figure S4. The comparison of gene expression pattern between high confidence genes and annotated genes in chicken. Figure S5-S15. The alignment of validated genes (CNOT3, HCFC1, KDM6B, PTGIR, GNG8, SLC7A8, CEBPE, RASSGRP4, FBXL19, BCL7C). (DOCX 2489 kb) [file 12864_2018_5407_MOESM2_ESM.docx]

**Supplementary data**

**Revisiting avian ‘missing’ genes from *de novo* assembled transcripts**

**Authors:**

Zhong-Tao Yin^1^, Feng Zhu^1^, Fang-Bin Lin^1^, Ting Jia^2^, Zhen Wang^1^, Dong-Ting Sun^2^, Guang-Shen Li^1^, Cheng-Lin Zhang^2^, Jacqueline Smith^3^, Ning Yang^1^, Zhuo-Cheng Hou^1^

**Affiliations:**

*^1^ National Engineering Laboratory for Animal Breeding, Key Laboratory of Animal Genetics, Breeding and Reproduction of the Ministry of Agriculture, College of Animal Science and Technology, China Agricultural University, Beijing, China 100193;*

***^2^*** *Beijing Key Laboratory of Captive Wildlife Technologies, Beijing Zoo, Beijing, 100044, China.*

*^3^ The Roslin Institute & R(D)SVS, University of Edinburgh, Easter Bush, Midlothian, EH25 9RG, UK.*

**Table S11 List of primers used for PCR validation**

| Gene Name | Primers’ Sequence(5’to 3’) | | Amplicon  (bp’s) | Temperature (℃) | |
| --- | --- | --- | --- | --- | --- |
|  | Forward | Reverse |  | Forward | Reverse |
| CNOT3  HCFC1  KDM6B  RASGRP4  FBXL19  BCL7C  PTGIR  GNG8  SLC7A8  CEBPE | GGTGCTGCTGGACTGGTTGAAG  CCACCACCATCGGCAACAAGAT  CGCACACCACCATCGCCAAGTA  TTCTGCGACAACTGTGCTGGAT  GGCAGTGTATCGCAGTGAAGGA  GCGACGAGCAATCTCTGATGGA  CCTTCTCTGCTGCCTTCTGCTC  AGCAGCACCATGAGCATCGG  GCCTGTGCCATCATCATCGGTA  GCGACATCTGCGAGAACGAGAA | AGATCGACCGCTGCCTGAAGAA  TGTCCTCCAGCGTGTCCATCA  TGAACACCTCCACGTCGCACTC  TCGGTCCTTGCTGCCTTCATTG  CTCATTGGCAAGGCTGTTCTCG  GAACTGGAGAGGCTGAGCAAGG  GGTTGCTGATGACGCTGAGGTT  CAGAGCAGGGCGCAGAAGAAT  CCTGAATGCGTGTTGCCCATC  CTGCTGCTTGCTGTGCTGGAA | 752  171  930  279  395  469  219  206  430  107 | 60.0  59.5  61.7  58.8  58.7  58.8  59.3  58.8  58.8  59.4 | 60.1  59.3  61.1  59.5  58.2  58.1  59.5  58.9  58.4  59.9 |


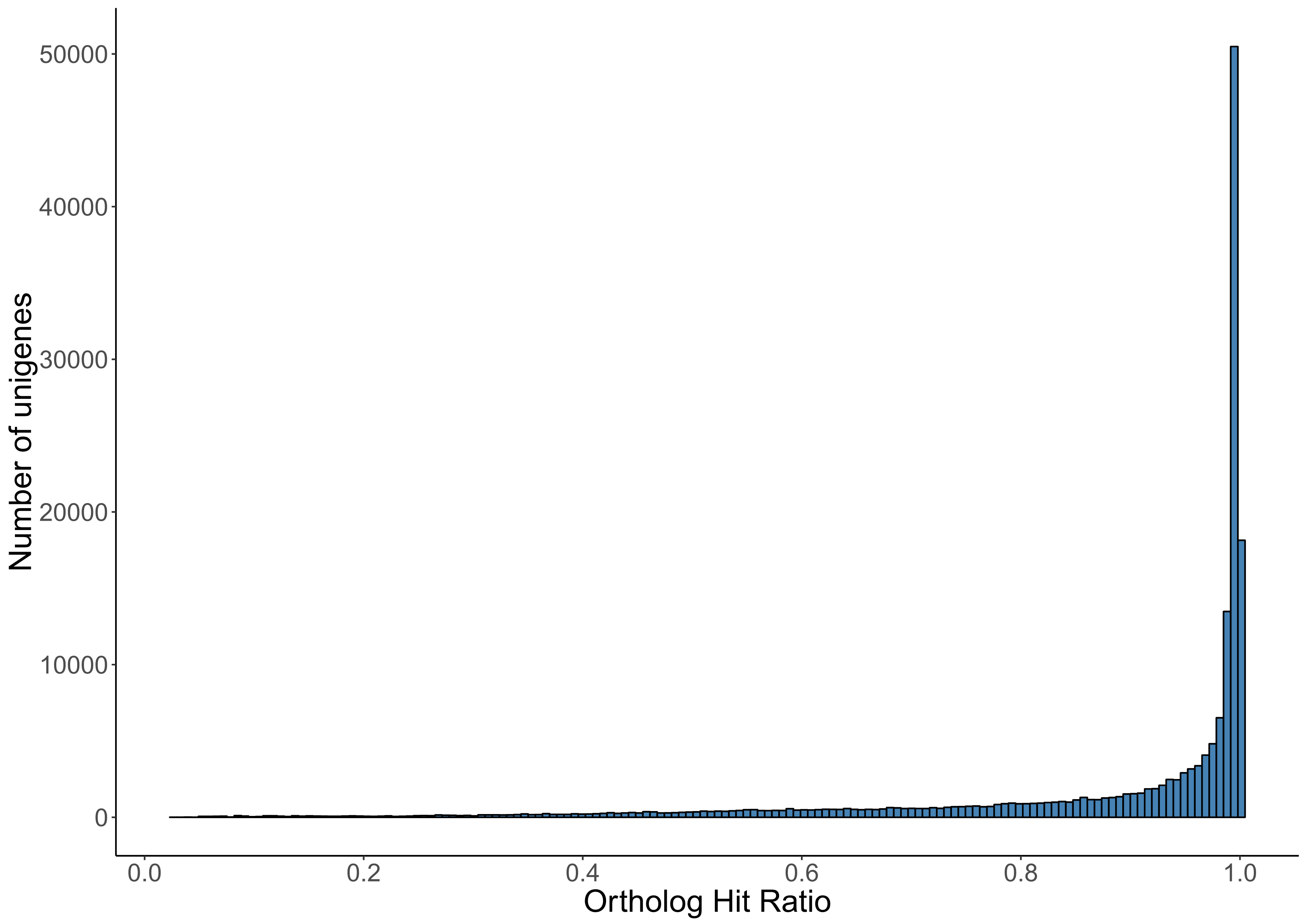
**Figure S1: Distribution of orthology hit ratio of assembly transcripts using chicken annotation (GalGal5) as the reference with which to compare *de novo* assembled transcripts**

**Figure S2: Distribution of recovered missing genes on chicken chromosomes**

**Figure S3: Co-linear analysis of chicken-human missing syntenic blocks**


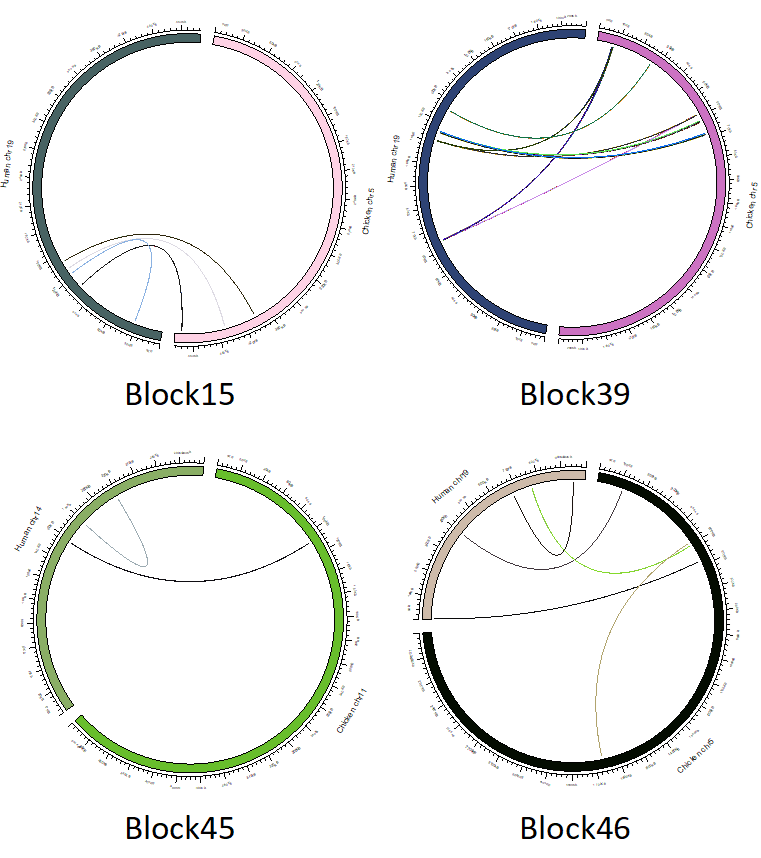


**Figure S4: The comparison of gene expression pattern between high confidence genes and annotated genes in chicken.**

(A) The percentage of genes that expressed most highly in each tissue both in high-confidence genes and annotated genes. (B) The comparison of the percentage of expressed genes in each tissue between high-confidence genes and annotated genes.

**Figure S5: Alignment of BCL7C**

**Figure S6: Alignment of CEBPE**

**Figure S7: Alignment of CNOT3**

**Figure S8: Alignment of** **FBXL19**

**Figure S9: Alignment of** **GNG8**

**Figure S10: Alignment of** **SLC7A8**

**Figure S11: Alignment of** **RASGRP4**

**Figure S12: Alignment of** **PTGIR**

**Figure S13: Alignment of** **HCFC1**

**Figure S14: Alignment of** **HCFC1**

**Figure S15: Alignment of** **KDM6B**
